# A synthetic combinatorial approach to disabling deviant Hedgehog signaling

**DOI:** 10.1038/s41598-018-19408-9

**Published:** 2018-01-18

**Authors:** C-W. Fan, N. Yarravarapu, H. Shi, O. Kulak, J. Kim, C. Chen, L. Lum

**Affiliations:** 1 Department of Cell Biology, University of Texas Southwestern Medical Center, Dallas, TX 75390 USA; 2 Department of Biochemistry, University of Texas Southwestern Medical Center, Dallas, TX 75390 USA; 3 Department of Internal Medicine, University of Texas Southwestern Medical Center, Dallas, TX 75390 USA; 4 Present Address: Pfizer Worldwide Research and Development, 10724 Science Center Drive, La Jolla, CA 92121 USA

## Abstract

Mutations in components of the Hedgehog (HH) signal transduction pathway are found in the majority of basal cell carcinoma (BCC) and medulloblastoma incidents. Cancerous cells with intrinsic or acquired resistance to antagonists targeting the seven transmembrane effector Smoothened (SMO) frequently invoke alternative mechanisms for maintaining deviant activity of the GLI DNA binding proteins. Here we introduce a chemical agent that simultaneously achieves inhibition of SMO and GLI activity by direct targeting of the SMO heptahelical domain and the GLI-modifying enzymes belonging to the histone deacetylase (HDAC) family. We demonstrate a small molecule SMO-HDAC antagonist (IHR-SAHA) retains inhibitory activity for GLI transcription induced by SMO-dependent and -independent mechanisms frequently associated with cancer biogenesis. Synthetic combinatorial therapeutic agents such as IHR-SAHA that a priori disable cancer drivers and anticipated mechanisms of drug resistance could extend the duration of disease remission, and provide an alternative clinical development path for realizing combinatorial therapy modalities.

## Introduction

Cellular response to the secreted HH proteins is initiated upon their binding to the multi-pass protein Patched 1 (PTCH1), a suppressor of the seven transmembrane receptor Smoothened (SMO)^1^. Activated SMO promotes SUFU disassociation from the GLI DNA binding proteins thus licensing them for gene transcriptional activation^2,3^. Deviant HH pathway activity associated with several cancers including medulloblastoma (MB) and basal cell carcinoma (BCC) is commonly induced by mutations in *PTCH1*^4,5^. SMO antagonists that are FDA-approved for the management of metastatic BCC (Vismodegib and Sonidegib) are able to restore homeostatic levels of signaling and blunt tumor growth^6^.

Despite an impressive initial response in some metastatic BCC patients, durable tumor growth suppression by SMO antagonists has been elusive and few treatment options that are available to patients after progression. Yet, the majority of the tumors that re-emerge are likely to be still dependent upon GLI transcriptional activity as determined by the appearance of mutations in SMO that prevent drug binding^7–11^, kinase-dependent mechanisms promoting sustained GLI activity in the absence of SMO input^12,13^, or *GLI2* gene amplification^8,14^. Thus, agents that disrupt GLI activity have broader indications than those targeting SMO in HH-associated cancers particularly in cases of drug resistance.

A number of strategies for disrupting GLI activity have been evaluated including those that promote GLI protein turn-over such as arsenic trioxide^15,16^ or GANT61^17^, instigate SUFU activity (ABT-199)^18^, or have limited mechanistic accounting^19^. The activity of GLI proteins also appear to be blunted by their acetylation thus offering opportunities for disabling GLI activity by blocking GLI deacetylases^20^. This strategy appears to be useful in blocking the growth of medulloblastomas in preclinical models of the disease^21^.

We had previously described a symmetric molecule with potent SMO inhibitory activity called IHR-1^22^. During the course of generating an fluorophore-labeled probe for visualizing IHR-1 interaction with SMO, we identified an active intermediate containing a long aliphatic linker that retained similar activity to the parental compound. We recognized that with an additional chemical step one could install the histone deacetylase (HDAC)-inhibitory pharmacoperones found in suberanilohydroxamic acid (SAHA, also known as Vorinostat) to potentially generate a dual antagonist. Here we characterize the mechanism of action for this molecule called IHR-SAHA that supports HH pathway inhibitory activity.

## Results

### Generation of a SMO-HDAC antagonist

**Figure 1:**
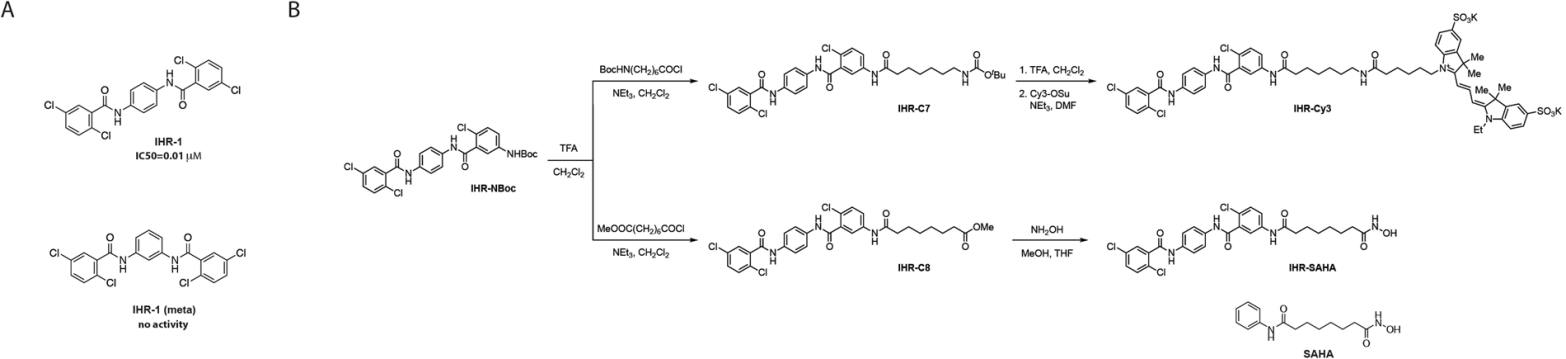
The origin of IHR-SAHA, a fusion molecule with potentially dual cellular activities. (**A**) Structures of IHR-1 and the inactive variant IHR-1 (meta)^22^. (**B)** The synthesis of IHR-Cy3 and IHR-SAHA. IHR-Cy3 is a chemical probe for monitoring IHR-1 interaction with SMO. Its synthetic intermediates IHR-NBoc and IHR-C7 retain anti-SMO activity (see Supplementary Fig. S1). The C7-amide moiety of IHR-C7 resembles SAHA and inspired the development of IHR-SAHA. The structure of SAHA is also shown.

The symmetric IHR-1 compound is a potent SMO antagonist identified from screening a diverse synthetic chemical library (Fig. 1A)^22^. Similar to other SMO antagonists, IHR-1 targets the heptahelical bundle to presumably promote an inactive conformation thus rendering cells HH-unresponsive. In addition, we had previously shown that the SMO inhibitory activity of IHR-1 is lost by switching the substitution pattern from *para* to *meta* (see Fig. 1A)^22^. The path to generating a fluorescent probe used for measuring IHR-1 binding to SMO (IHR-Cy3) entailed first replacing a chlorine atom of IHR-1 with an amino group followed by the addition of an aliphatic extension used to bridge Cy3 to IHR-1 (IHR-C7; Fig. 1B, Supplementary Fig. S1)^22^. The retention of anti-SMO activity in IHR-Cy3 suggests that chemical adducts with other cell biological activities in place of Cy3 could be engineered into this backbone^22^. To test this hypothesis, we created an IHR-1 derivative that now incorporates a molecule resembling the HDAC inhibitor SAHA (see Fig. 1B).

### IHR-SAHA retains HDAC inhibitory activity

**Figure 2:**
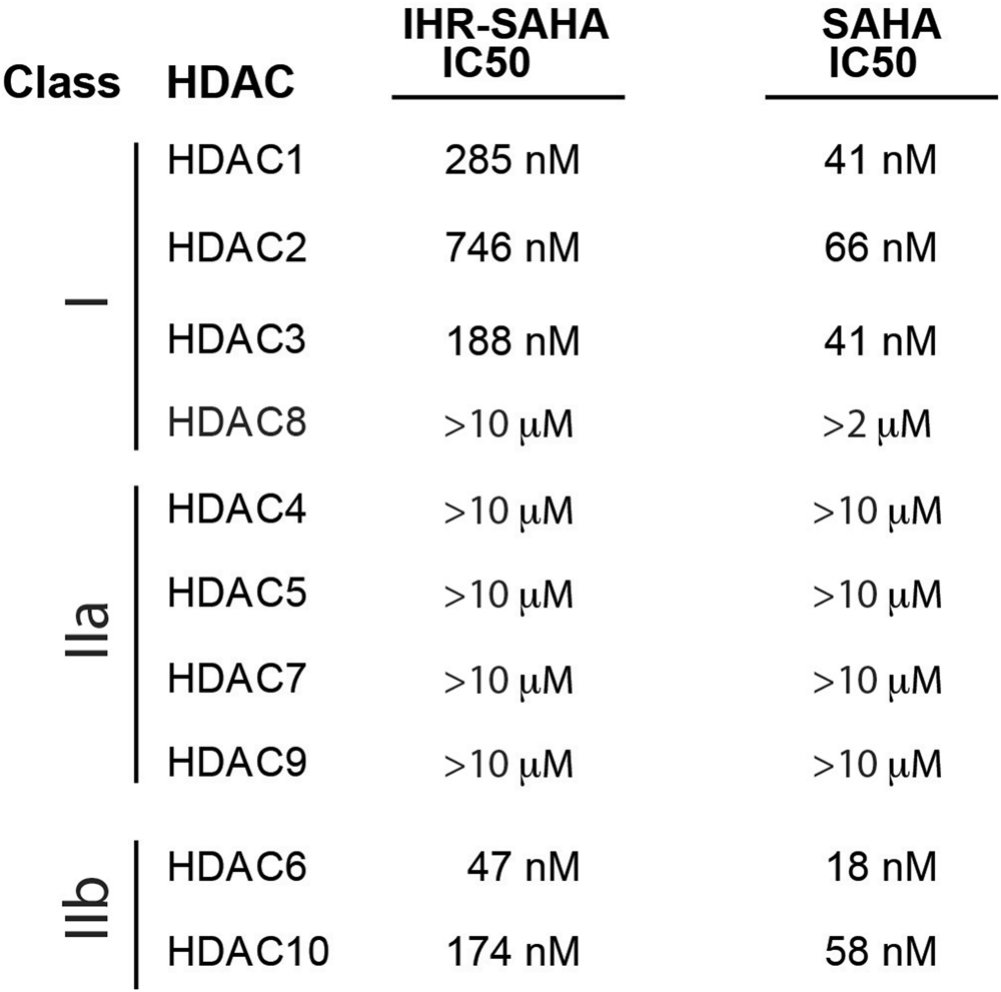
IHR-SAHA retains similar specificity for SAHA-targeted HDACs. Activity profile of IHR-SAHA against different classes of HDACs was evaluated using an *in vitro* deacetylation assay. Each data point used to generate the IC50 curve is an average of duplicate experiments (see Supplementary Table S1). SAHA activity in the same assay platform from a reference dataset is provided.

To determine if the addition of IHR-1 to SAHA altered its inhibitory profile amongst HDAC family members, we performed *in vitro* IC50 assays against purified HDAC proteins (Fig. 2**;** Supplementary Table S1). Comparing these results with those previously generated using the same assay conditions and reagents^23^, we observed a similar activity profile suggesting that the addition of IHR-1 did not significantly change the selectivity of SAHA for class I and II HDAC family members (see Fig. 2). Based on the outcome of studies focused on the major HDAC classes known to be inhibited by SAHA^24^, we assume differences in any biological activity between SAHA and IHR-SAHA are not likely to be greatly impacted by alterations in the selectivity of HDAC inhibition.

### SMO and HDAC inhibitory activities in IHR-SAHA are modular

**Figure 3:**
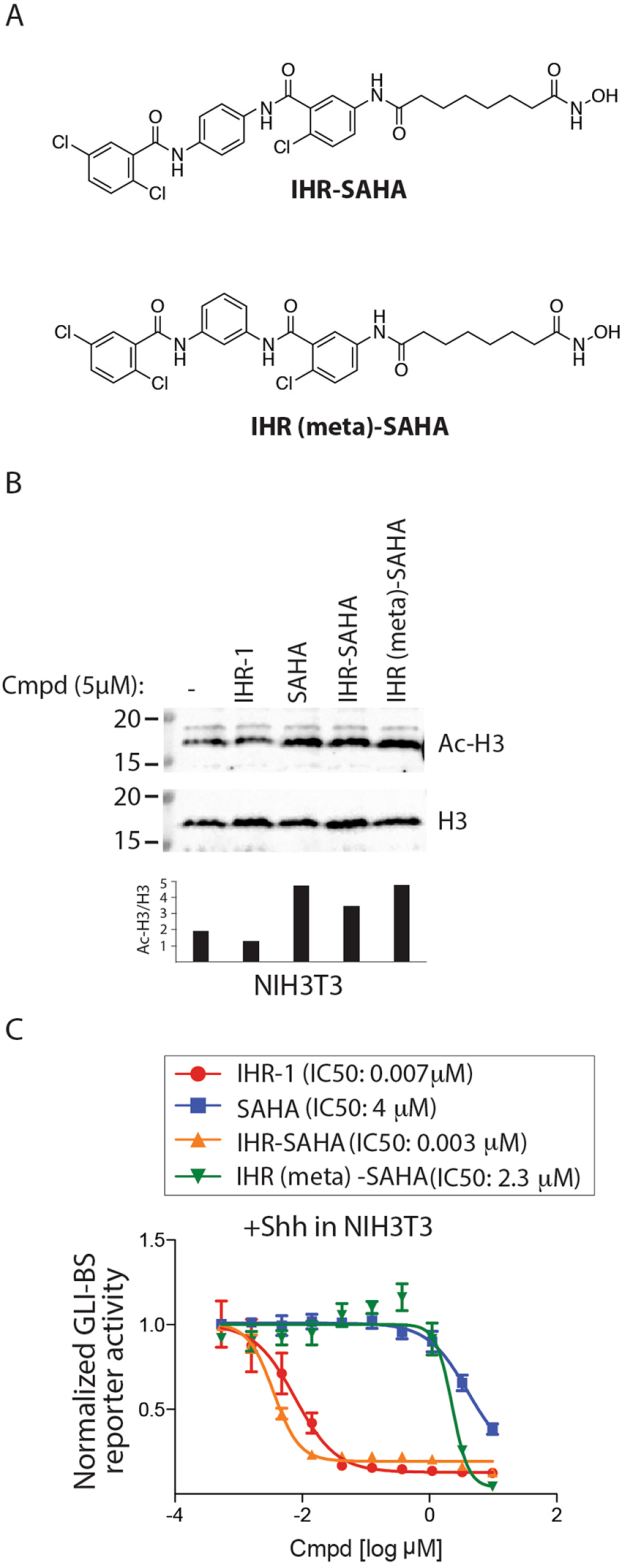
Anti-SMO and -HDAC activities in IHR-SAHA are modular. (**A**) Structures of IHR-SAHA and IHR (meta)-SAHA. IHR (meta)-SAHA is a fusion of IHR-1 (meta) and SAHA. (**B**) IHR-SAHA and IHR (meta)-SAHA retain HDAC inhibitory activity as measured by Western blotting for acetylated histone 3 (Ac-H3) in NIH-3T3 cells. The ratio of Ac-H3 to total H3 was quantified. Unprocessed blots are found in Supplementary Fig. S3. Two independent experiments were performed. (**C**) IHR-SAHA and IHR (meta)-SAHA exhibit different levels of anti-HH pathway activity. Indicated compounds were evaluated for their activity using a HH pathway reporter (GLI-BS reporter). Pathway response is reported as the ratio of Gli-BS and control reporter activities. Data show the mean and SD of three samples. Two independent experiments were performed.

To determine if the activity of the anti-SMO or anti-HDAC warheads is influenced by their chemical linkage, we generated an IHR (meta)-SAHA molecule which presumably would allow us to evaluate the anti-HDAC activity of IHR-SAHA in the absence of anti-SMO activity (Fig. 3A). Inactivation of the anti-SMO activity in IHR (meta)-SAHA did not affect the ability of IHR (meta)-SAHA to block nuclear HDAC activity as evidenced by the accumulation of acetylated-histone 3 (Ac-H3) suggesting that the two chemical activities are uncoupled (Fig. 3B). In addition, IHR (meta)-SAHA retains the ability to block SHH-induced GLI activity despite not possessing anti-SMO activity when evaluated using a cell based reporter assay of HH signaling (Fig. 3C).

### IHR-SAHA exhibits activity against drug-resistant drivers of GLI activity

**Figure 4:**
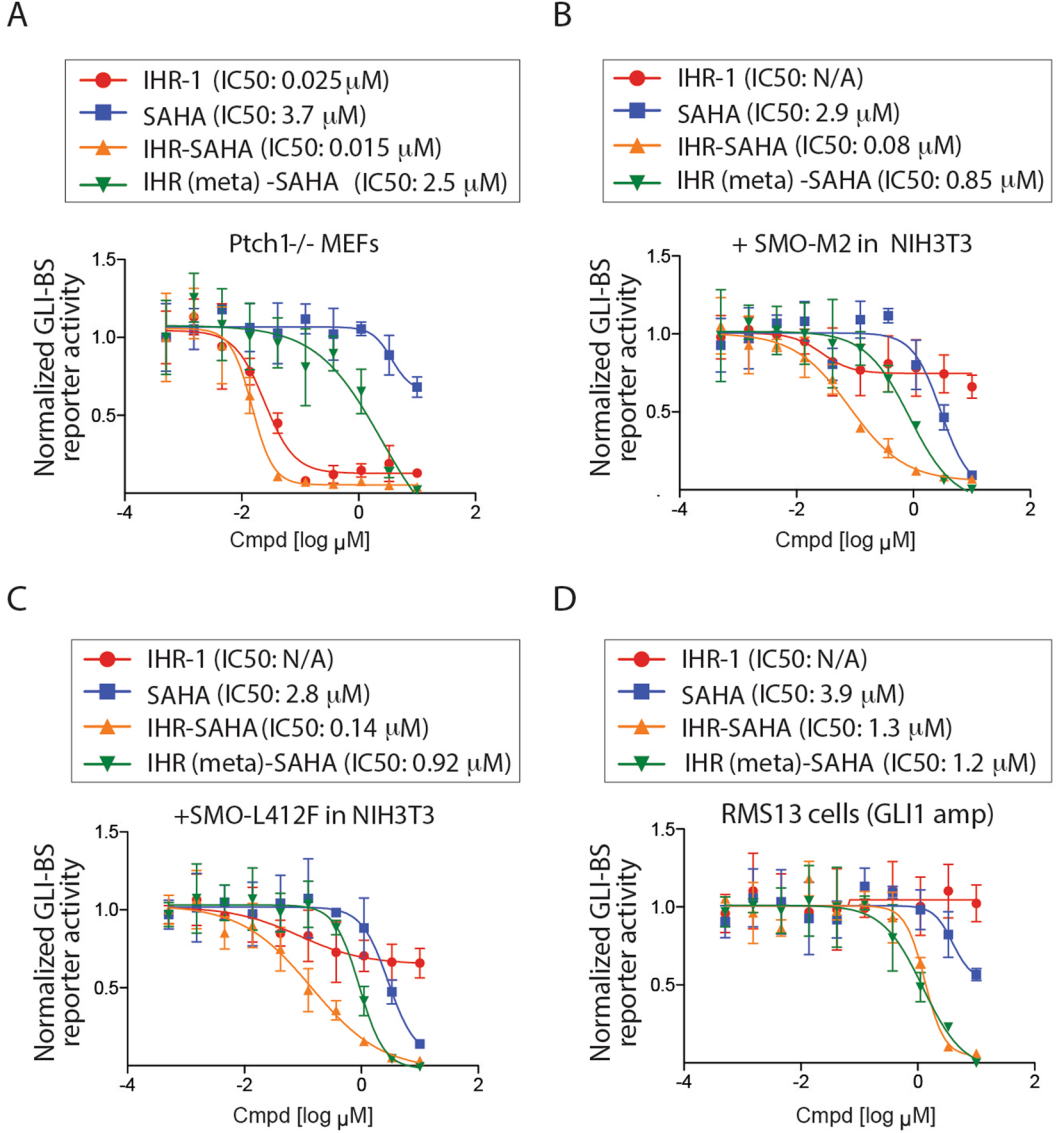
Activity of IHR-SAHA against drug resistance forms of HH signaling. GLI-BS and control reporter were transfected into *Ptch*^−/−^ MEFs (**A**), NIH3T3 cells with SMO-M2 DNA (**B**) or SMO-L412F DNA (**C**), or RMS13 cells which harbor *GLI1* gene amplification (**D**). Pathway response is reported as the ratio of Gli-BS and control reporter activities. Data show the mean and SD of three samples. Two independent experiments were performed.

We next examined the activity of IHR-SAHA against three cancer-related genetic alterations known to induce deviant GLI activity (Fig. 4A–C). Loss of *PTCH1* is associated with ~70% of BCCs and 45% of SHH-subtyped medulloblastomas whereas the *SMO* mutations (such as the constitutively active SMO-M2 [W535L) mutation] is less frequently found in these diseases^4,5^. SMO-L412F is an acquired SMO mutation that alters the drug-binding pocket in the heptahelical domain^14^. In cells with loss of *Ptch1*, IHR-SAHA exhibited slightly improved levels of activity against SMO-driven GLI activity compared with IHR-1 perhaps due to the attachment of SAHA to IHR-1. However, in cells expressing SMO-M2 or SMO-L412F which are intrinsically resistant to SMO antagonists^25^, IHR-SAHA exhibited a much greater activity compared to IHR-1. The different activity of IHR-1 and IHR-SAHA in the two contexts (*Ptch1* null and *SMO* activating mutations) could be explained by an increase in cell permeability of IHR-SAHA compared with IHR-1 (Supplementary Fig. S2) and the previously described rogue activity of SMO-M2 and SMO-L412F from an intracellular compartment that renders cell impermeable SMO antagonists less active^22^. In the case of *Ptch1* null cells, IHR-1 and IHR-SAHA exhibit similar ability to block GLI transcriptional activity due to SMO signaling activity from the primary cilium and not an intracellular component. We also more directly evaluated the effectiveness of IHR-SAHA to disable deviant GLI activity in cells harbor a *GLI* gene amplification (Fig. 4D). We observed anti-GLI activity with both IHR-SAHA and IHR (meta)-SAHA in RMS13 cells, which exhibit GLI-BS reporter activity as a consequence of *GLI1* amplification. We noted that SAHA appeared to be weaker in activity in this cell line compared to the IHR-SAHA or IHR (meta)-SAHA fusion molecules suggesting that the IHR-1 and IHR-1 (meta) adduct somehow improved SAHA activity in cultured cells.

### IHR confers increased HDAC activity to SAHA

**Figure 5:**
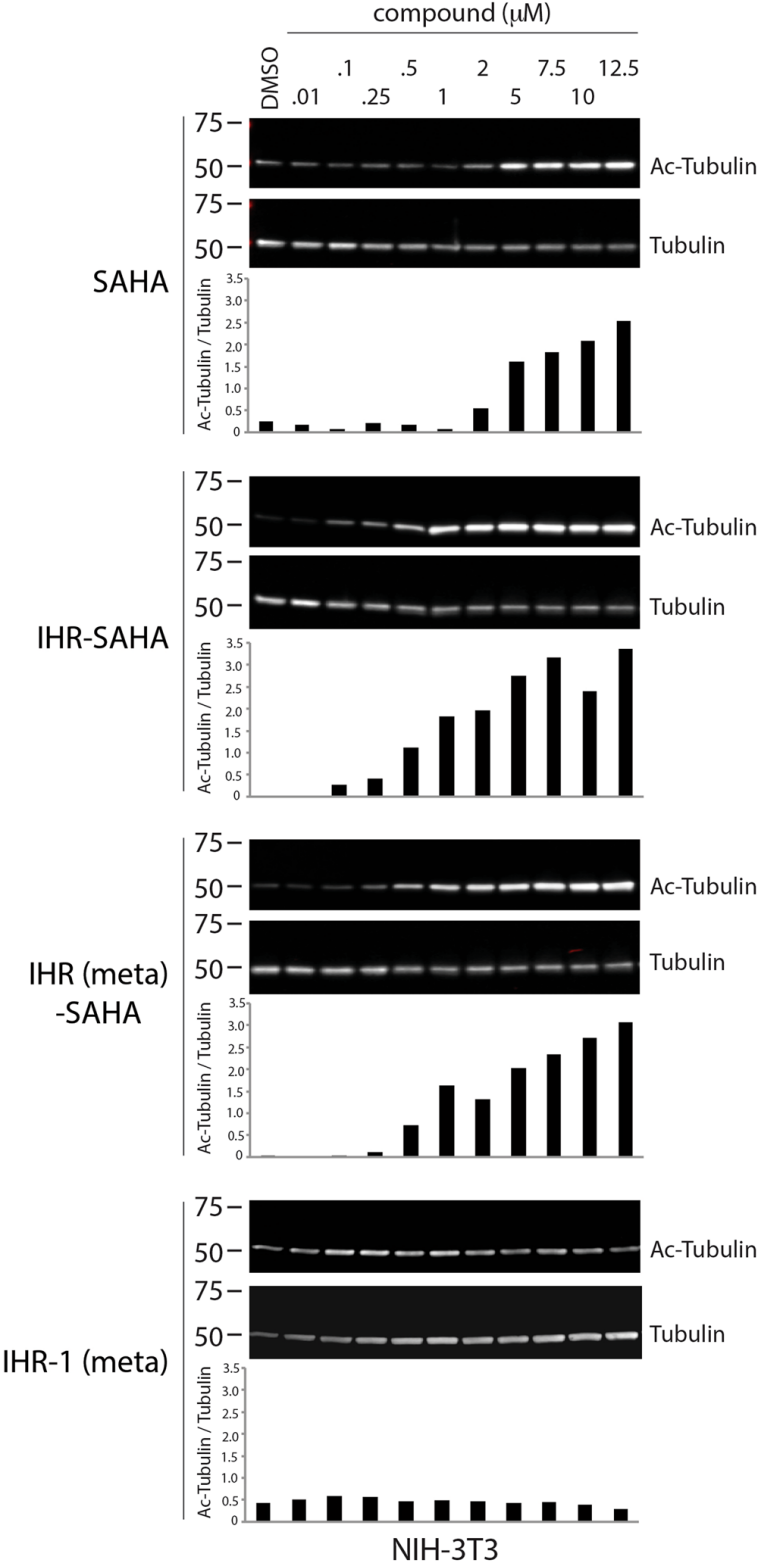
IHR-SAHA compounds exhibit increased HDAC inhibitory activity compared to SAHA. Abundance of total or acetylated tubulin was determined by Western blot analysis of NIH-3T3 cells treated with indicated concentrations of compound. The ratio of acetylated tubulin to total tubulin was quantified for each condition. Unprocessed blots are found in Supplementary Fig. S3. Two independent experiments were performed.

Consistent with increased activity of IHR-SAHA and IHR (meta)-SAHA fusion molecules compared with SAHA for inhibiting GLI activity seen in RMS13 cells which harbor *GLI1* amplification, we observed a similar trend for these molecules when using acetylated tubulin accumulation as a readout of SAHA activity (Fig. 5). Thus, the presence or absence of anti-SMO in the chemical agent does not appear to explain the increase in anti-HDAC activity seen in IHR-SAHA and IHR (meta)-SAHA (see Fig. 5). HDAC6, a target of SAHA, regulates the abundance of acetylated tubulin^26,27^. Though not evaluated here, the addition of IHR-1 may have increased the cell permeability of SAHA (just as SAHA may have increased the cell permeability of IHR-1) given that both IHR-SAHA and SAHA appear to exhibit similar activities against recombinant HDAC6 *in vitro* (see Fig. 2).

### IHR-SAHA prevents GLI binding to DNA

**Figure 6:**
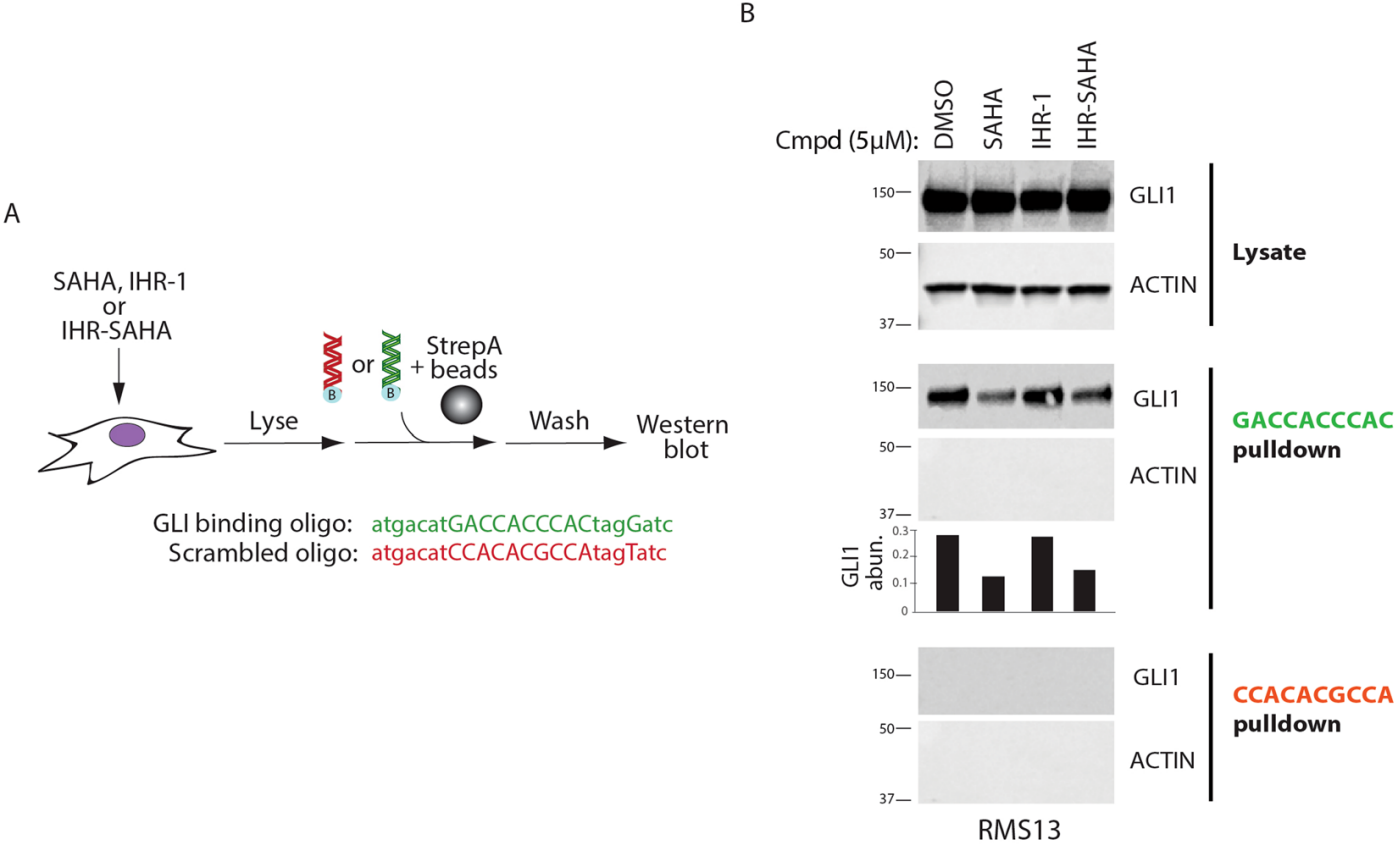
IHR-SAHA blocks GLI binding to DNA. **(A)** A biochemical assay for monitoring GLI interaction with DNA. Lysate from a cell line expressing a GLI protein is incubated with biotinylated oligos encoding either a consensus GLI binding motif or a scrambled sequence. The abundance of GLI binding to DNA is determined by Western blot analysis of material associated with oligo-bound streptavidin sepharose. (**B**) SAHA and IHR-SAHA decrease GLI binding to DNA. Lysate from RMS13 cells treated with indicated compounds were subjected to the assay described in (**A**) “GACCACCCAC” (green) is a GLI binding site oligonucleotide whereas “CCACACGCCA” (red) is a scrambled binding site oligonucleotide. The abundance of GLI1 bound to DNA was quantified. Unprocessed blots are found in Supplementary Fig. S3. Two independent experiments were performed.

Whereas GLI1 acetylation has previously been shown to be sensitive to a chemical inhibitor of HDACs, the effect of such compounds on GLI binding to DNA has not been interrogated^20^. We developed an *in vitro* assay that would allow us to monitor the effects of IHR-SAHA on GLI1 interaction with DNA (Fig. 6A**)**. Monitoring endogenously expressed GLI1 protein in RMS13 cells, we observed loss of GLI1 binding to solid-support immobilized oligos harboring a consensus GLI binding sequence in the presence of SAHA and IHR-SAHA but not IHR-1 (Fig. 6B). Thus, the acetylation status of GLI proteins likely influences their ability to bind DNA and to regulate transcription of HH-controlled target genes.

## Discussion

A testament to the growth-promoting prowess of GLI proteins in certain cancer types such as basal cell carcinoma and medulloblastoma is the continued reliance of cancerous cells resistant to SMO antagonists on GLI activity^5^. A number of strategies to counter the emergence of drug resistance have been proposed including those targeting PI3K^8,13,28,29^ or atypical protein kinase C ι/λ (aPKC-ι/λ)^12^ which promote GLI activity even in the absence SMO signal, or inducing SUFU activity by removing the suppressive interaction of cytosolic prosurvival BCL-2 family members with SUFU^18^. A priori treatment with dual pathway antagonists such as the one described here may delay the emergence of drug resistance facilitated by these previously observed mechanisms. We also note that a similar strategy to the one described here has been reported albeit this case using the SMO antagonist Vismodegib as a scaffold^30^ or as a drug combination^31^. Vorinostat is FDA-approved for the treatment of cutaneous T cell lymphoma, but so far has shown a more limited response in solid tumors^32^. Given the contribution of HH signaling in some of these diseases, there may be indications for the use of IHR-SAHA in cancers not routinely sequenced for mutations in known HH pathway components.

The improvements in activity of both IHR-1 and SAHA from their chemical fusion reinforce a lesson learned from our previous study that the activity changes associated with altered intrinsic cell membrane permeability of chemical probes could be exploited for understanding the subcellular site-of-action for protein queries^22^. The addition of SAHA likely increased the cell membrane permeability of IHR-1 as has been seen with other IHR-1 derivatives that improves its ability to target intracellularly localized SMO-M2 and SMO-L412F^22^ and with cell permeability studies using a molecule similar to IHR-SAHA (see Supplementary Fig. S2). We also acknowledge that differences in the cell membrane permeability of these molecules are not mutually exclusive from hypotheses that include IHR-SAHA inhibiting SMO mutants in a manner distinct from that of IHR-1 alone, or that IHR-SAHA simply inhibits SMO heptahelical domain more potently than unmodified IHR-1. Regardless of the mechanism, the observed increased inhibition of SMO and HDACs with IHR-SAHA when compared with either individual IHR-1 or SAHA that may correlate with improvements in the *in vivo* activity of both molecules as a consequence of their chemical fusion.

HDAC1 and HDAC6 activities are inhibited by IHR-SAHA at a nanomolar range *in vitro*. Whereas HDAC1 inhibition has been shown to directly affect GLI acetylation^20^, genetically based elimination or chemical inhibition of HDAC6 with selective small molecules is sufficient to block a subset of GLI transcriptional targets and SMO-M2-driven medulloblastoma growth *in vivo*^33^. In addition, HDAC6 inhibitors modulate primary cilium stability, an organelle that facilitates the relinquishing of SUFU suppression^2,34^ and proteolytic processing of GLI3 into a transcriptional repressor^35–39^. Thus, IHR-SAHA could impact GLI activity directly by targeting HDAC1 and indirectly by altering aspects of primary cilium function.

In addition to the cancer biology and medicinal chemistry considerations that may argue for a utility of multi-targeting agents in cancer management, there may also exist advantages for such a strategy in clinical development stages. For phase I studies with two drug combinations, optimal dose schedules for each drug need to be identified through formal dose escalation studies^40^ where the concentration of one drug is increased at various fixed concentrations of the other drug. These studies require several treatment arms, larger number of patients and increased time compared to single drug phase I studies. Furthermore, pharmacokinetics of each drug will need be monitored as the drug-drug interactions may alter their metabolism and bioavailability^40^. With single fusion compounds, such as IHR-SAHA, the dose of each drug component is fixed thus requiring a simple dose escalation study and only the single fusion compound need to be monitored for pharmacokinetics. Thus, fusion compounds can potentially accelerate and simplify the clinical development of drugs. Future studies using *in vivo* tumor models will evaluate the utility of such multi-targeting agents for overcoming the plasticity of cancer cell signaling networks that enable drug resistance.

## Methods

### Cell culture and chemical reagents

NIH3T3 and RMS13 cell lines were purchased from ATCC. *Ptch1*^−/−^ MEFs and 3T3-ShhFL cell lines were previously described^22^. SAHA was purchased from Cayman Chemical (#10009929). All other compounds were synthesized at UT Southwestern (see below).

### Chemical synthesis

#### Synthesis of IHR-SAHA

A solution of *tert*-butyl (4-chloro-3-((4-(2,5-dichlorobenzamido)phenyl)carbamoyl)phenyl)carbamate^22^ (2.0 g, 3.7 mmol) in trifluoroacetic acid/dichloromethane (20% v/v, 6 mL) was stirred at 23 °C for 20 min. The volatiles were then removed and the residue purified by silica gel column chromatography (ethyl acetate/hexanes = 1/2 to 1/1) to give 5-amino-2-chloro-*N*-(4-(2,5-dichlorobenzamido)phenyl)benzamide as a yellow solid (1.5 g, 93% yield). ^1^H NMR (500 MHz, DMSO-*d*_6_) δ 10.58 (s, 1H), 10.39 (s, 1H), 7.75 (d, *J* = 2.3 Hz, 1H), 7.68 (q, *J* = 8.7 Hz, 6H), 7.64–7.59 (m, 3H), 7.14 (d, *J* = 8.6 Hz, 1H), 6.70 (d, *J* = 2.7 Hz, 1H), 6.65 (dd, *J* = 8.6, 2.6 Hz, 1H), 5.49 (brs, 2H). MS (ESI)^+^ calcd for C_20_H_14_Cl_3_N_3_O_2_ [M + H]^+^ 434.0, found 434.0.

To a solution of monomethyl suberate (0.67 g, 3.63 mmol) in a mixture of dichloromethane (9 mL) and *N*,*N*-dimethylformamide (0.1 mL) was added oxalyl chloride (0.37 mL, 4.36 mmol) at 0 °C. After stirring at 23 °C for 10 min, a mixture of 5-amino-2-chloro-*N*-(4-(2,5-dichlorobenzamido)phenyl)benzamide (1.5 g, 3.45 mmol) and triethylamine (1.52 mL, 10.9 mmol) in dichloromethane (3 mL) was added and the solution was stirred at 23 °C for 3 h. After removing the volatiles, the residue was dissolved in methanol (12 mL) and aqueous hydroxylamine (50%, 1.2 mL). The solution was stirred at 60 °C for 16 h before concentrated and triturated with ethyl acetate/hexanes (1/1, 20 mL) to give IHR-SAHA as a white solid (1.0 g, 48% yield). ^1^H NMR (500 MHz, DMSO-*d*_6_) δ 10.60 (s, 1H), 10.53 (s, 1H), 10.35 (s, 1H), 10.19 (s, 1H), 8.69 (brs, 1H), 7.84 (d, *J* = 2.5 Hz, 1H), 7.76 (d, *J* = 2.3 Hz, 1H), 7.74–7.66 (m, 5H), 7.66–7.57 (m, 2H), 7.49 (d, *J* = 8.7 Hz, 1H), 2.33 (t, *J* = 7.3 Hz, 2H), 1.94 (t, *J* = 7.3 Hz, 2H), 1.63–1.55 (m, 2H), 1.52–1.46 (m, 2H), 1.35–1.22 (m, 4H). MS (ESI)+ calcd for C28H27Cl3N4O6 [M + H]+ 605.1, found 605.0.

#### Synthesis of IHR (meta)-SAHA

A solution of *tert*-butyl (4-chloro-3-((3-(2,5-dichlorobenzamido)phenyl)carbamoyl)phenyl)carbamate^22^ (3.1 g, 6.2 mmol) in trifluoroacetic acid/dichloromethane (20% v/v, 20 mL) was stirred at 23 °C for 20 min. The volatiles were then removed and the residue purified by silica gel column chromatography (ethyl acetate/hexanes = 1/2 to 1/1) to give 5-amino-2-chloro-*N*-(3-(2,5-dichlorobenzamido)phenyl)benzamide as a yellow solid (1.6 g, 59% yield). ^1^H NMR (400 MHz, DMSO-*d*_6_) δ 10.61 (s, 1H), 10.42 (s, 1H), 8.16 (t, *J* = 2.1 Hz, 1H), 7.79–7.67 (m, 1H), 7.65–7.52 (m, 2H), 7.43 (d, *J = *7.9 Hz, 1H), 7.41–7.35 (m, 1H), 7.28 (d, *J* = 7.9 Hz, 1H), 7.10 (d, *J* = 8.4 Hz, 1H), 6.74–6.58 (m, 2H), 5.44 (brs, 2H). MS (ESI)^+^ calcd for C_20_H_14_Cl_3_N_3_O_2_ [M + H]^+^ 434.0, found 434.0.

To a solution of monomethyl suberate (0.53 g, 2.81 mmol) in a mixture of dichloromethane (20 mL) and *N*,*N*-dimethylformamide (0.1 mL) was added oxalyl chloride (0.3 mL, 3.37 mmol) at 0 °C. After stirring for 10 min at 23 °C, a mixture of 5-amino-2-chloro-*N*-(3-(2,5-dichlorobenzamido)phenyl)benzamide (1.11 g, 2.56 mmol) and triethylamine (1.18 mL, 8.43 mmol) in dichloromethane (10 mL) was added and the solution was stirred at 23 °C for 3 h. After removing the volatiles, the residue was dissolved in methanol (10 mL) and aqueous hydroxylamine (50%, 1.7 mL). The solution was stirred at 60 °C for 16 h before concentrated and triturated with ethyl acetate/hexanes (1/1, 15 mL) to give IHR (meta)-SAHA as a yellow solid (420 mg, 27% yield). ^1^H NMR (400 MHz, DMSO-*d*_6_) δ 10.63 (s, 1H), 10.56 (s, 1H), 10.35 (s, 1H), 10.17 (s, 1H), 8.78–8.59 (m, 1H), 8.27–8.08 (m, 1H), 7.78 (d, *J* = 2.5 Hz, 1H), 7.73–7.62 (m, 2H), 7.56 (d, *J* = 4.2 Hz, 2H), 7.42 (dd, *J* = 14.9, 8.1 Hz, 3H), 7.30 (t, *J* = 8.0 Hz, 1H), 2.28 (t, *J* = 6.9 Hz, 2H), 1.91 (t, *J* = 6.8 Hz, 2H), 1.54 (m, 2H), 1.46 (m, 2H), 1.24 (m, 4H). MS (ESI)^+^ calcd for C_28_H_27_Cl_3_N_4_O_6_ [M + H]^+^ 605.1, found 605.0.

### ***In vitro*HDAC profiling**

HDAC profiling was performed at BPS Bioscience (San Diego, CA). All of the compounds are dissolved in DMSO. A series of dilutions of the compounds were prepared with 10% DMSO in HDAC assay buffer (#50031) and 5 µl of the dilution was added to a 50 µl reaction so that the final concentration of DMSO is 1% in all of reactions. All of the enzymatic reactions were conducted in duplicate at 37 °C for 30 mins, except that the enzyme reactions for HDAC11 were at room temperature for 3 hrs. All of the reactions were performed in a 50 µl mixture containing HDAC assay buffer, 5 µg BSA, an HDAC substrate [HDAC Substrate 3 (BPS number 50037) or HDAC Class 2a Substrate 1 (BPS number 50040)], an HDAC enzyme (#50051-11) and a test compound. After enzymatic reactions, 50 μl of HDAC Developer (#50030) was added to each well and the plate was incubated at room temperature for an additional 20 mins. Fluorescence intensity was measured at an excitation of 360 nm and an emission of 460 nm using a Tecan Infinite M1000 microplate reader. HDAC activity assays were performed in duplicates at each concentration. The fluorescent intensity data were analyzed using the computer software, Graphpad Prism. In the absence of the compound, the fluorescent intensity (Ft) in each data set was defined as 100% activity. In the absence of HDAC, the fluorescent intensity (Fb) in each data set was defined as 0% activity. The percent activity in the presence of each compound was calculated according to the following equation: %activity = (F − Fb)/(Ft − Fb), where F = the fluorescent intensity in the presence of the compound. The values of % activity versus a series of compound concentrations were then plotted using non-linear regression analysis of Sigmoidal dose-response curve generated with the equation Y = B + (T − B)/1 + 10((LogIC50 − X) × Hill Slope), where Y = percent activity, B = minimum percent activity, T = maximum percent activity, X = logarithm of compound and Hill Slope = slope factor or Hill coefficient. The IC50 value was determined by the concentration causing a half-maximal percent activity. SAHA IC50 data for individual HDACs was previously generated by BPS using similar assay conditions.

### Reporter assay

The HH-responsive firefly luciferase reporter (GLI-BS) and a control *Renilla* luciferase reporter (SV40-RL) were transfected into indicated cell lines using Effectene (Qiagen) either alone or with indicated DNAs. 24 hrs after transfection, cells were switched to low serum media (3% calf serum), and grown for another 48 hrs. in 5% CO_2_ in the presence/absence of compounds. 3T3-ShhFL cell line that stably expressing SHH and the two reporters were cultured in a similar fashion. FL and RL activities in lysate generated using Passive Lysis Buffer (Promega) were then assessed using the Dual-Luciferase kit (Promega) and a 96-well plate reading luminometer (BMG). The ratio of FL/RL was calculated and the averaged ratios from three replicates were reported.

### Acetylation assay

For analyzing acetylation status of histone 3 and tubulin, NIH3T3 cells were grown to confluence in 6-well plates. After 48hrs of treatment with indicated chemicals, cells were lysed in RIPA buffer or 1% NP40/phosphate buffered saline/protease inhibitors (SIGMA, #S8820), the lysate cleared using a microcentrifuge, then 6 × sample loading buffer (Bioland Scientific) added to the supernatant. Proteins were separated on SDS-PAGE (BioRad Criterion TGX Precast Gels). Antibodies used for analyzing the blots were: acetylated tubulin (SIGMA, #T6793), tubulin, acetyl-histone H3 (Lys23), and histone H3 (Cell Signaling Technology, #2125 S, #8848 and #9717, respectively). Chemiluminescence was detected using a Li-COR Odyssey Imaging System.

### GLI DNA binding assay

A detailed protocol for DNA binding assay was previously described^41^. Briefly, cells treated for 48 hrs with compounds were lysed in 1% NP40/phosphate buffered saline/protease inhibitors and cleared using a microcentrifuge. The lysate was then incubated with 0.5 µM of double-strand biotinylated oligonucleotides and 40 µl streptavidin-agarose bead suspension (Thermo Scientific, 20349) for 2 hrs. The streptavidin agarose beads are washed (3X) with 1% NP40/phosphate buffered saline/protease inhibitors and eluted with 2X Laemmli sample buffer. The supernatants are separated on SDS-PAGE and analyzed using western blotting. Antibodies were purchased from the following vendors: GLI1 (Cell Signaling Technology, #2534) and actin (SIGMA, #A5441).

### Compound cellular permeability

Caco-2 cell permeability assay was previously described^22^. Briefly, Caco-2 cells were grown to confluence in 12-well transwell plates. Culture medium was replaced with 10μM of compounds in DMEM/ 3% calf serum and incubated for 6hrs. Media from the top and bottom chamber were collected and diluted in DMEM/ 3% calf serum as indicated in the figure. Diluted media were added to confluent 3T3-ShhFL cells for 48hrs and measure the Hh pathway activity by reporter assay.

### Data availability

All data generated or analyzed during this study are included in this published article (and its Supplementary Information files).

## Electronic supplementary material

Supplementary figures S1-S3

Table 1
