# Supplementary material for: A synthetic combinatorial approach to disabling deviant Hedgehog signaling
Source: Sci Rep. 2018 Jan 18;8:1133. doi: 10.1038/s41598-018-19408-9 (PMC5773580; doi:10.1038/s41598-018-19408-9)
Supplement: Supplementary file 1 — Supplementary figures S1-S3 [file 41598_2018_19408_MOESM1_ESM.pdf]

A synthetic combinatorial approach to disabling deviant Hedgehog signaling

Fan C-W[1*], Yarravarapu N[1*], Shi H[2], Kulak O[1], Kim J[3], Chen C[2Δ+], and Lum L[1Δ+■]

Department of Cell Biology[1], Biochemistry[2], and Internal Medicine[3]
University of Texas Southwestern Medical Center, Dallas TX 75390

■current address:
Pfizer Worldwide Research and Development
10724 Science Center Drive, La Jolla, CA 92121

[Δ]Correspondence to: Chuo Chen (chuo.chen@utsouthwestern.edu) and Lawrence Lum (Lawrence.lum@utsouthwestern.edu)

* These authors contributed equally to this work
[+] These authors jointly supervised this work

Supplementary Figure S1

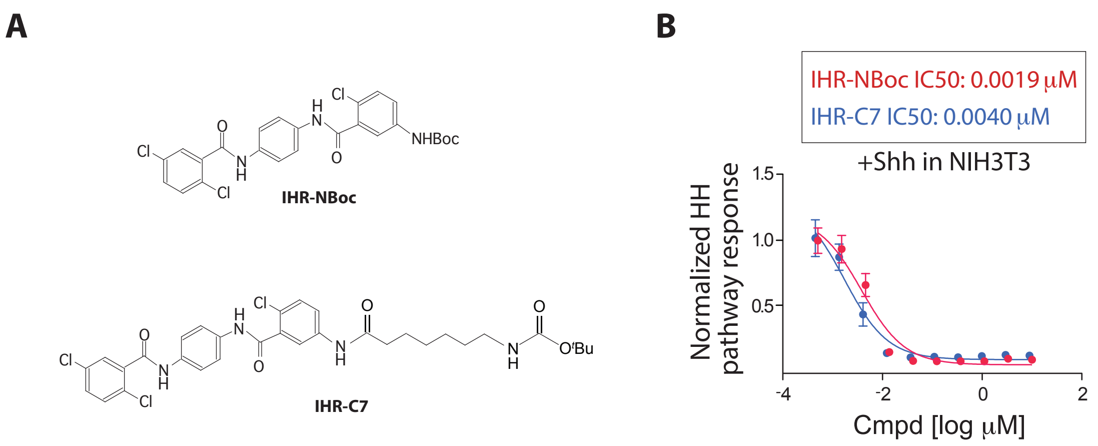

Supplementary Figure S1. IHR-NBoc and IHR-C7 retain anti-Smo activity. A. Structures of IHR-NBoc and IHR-C7. **B.** IHR-NBoc and IHR-C7 exhibit similar anti-HH pathway activity. Indicated compounds were evaluated for their activity using a HH pathway reporter (GLI-BS reporter). Data show the mean and SD of three samples. Two independent experiments were performed.

Supplementary Figure S2

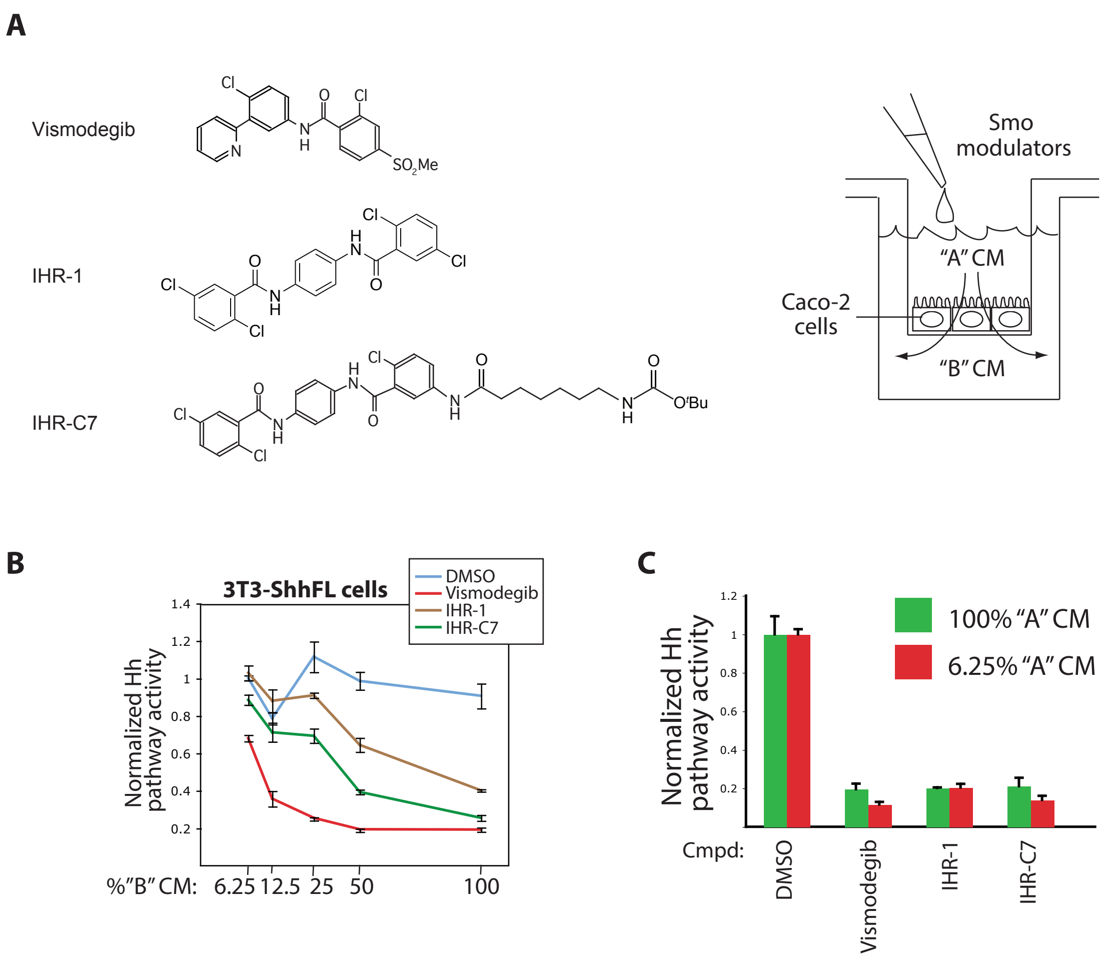

Supplementary Figure S2. IHR-C7 exhibits improved ability to transverse a cell monolayer. **A.** Schematic of a transwell assay to measure cellular permeability of small molecule modulators of Smo activity. Smo antagonists shown at left are deposited in a growth chamber that is separated into two chambers by a monolayer of Caco-2 cells and a porous membrane (0.4μm). Vismodegib is a FDA approved Smo antagonist for treating basal cell carcinoma. CM stands for conditioned medium. **B.** Medium from the lower ("B") chamber was collected 6hrs later and diluted as indicated to test for Hh inhibitory activity by using 3T3-ShhFL cells. Compound with better cell membrane permeability has higher compound concentration in the "B" CM thus inhibit Hh pathway activity at a greater extent. Data show the mean and SD of three samples. Two independent experiments were performed. **C.** Medium from chamber A was applied to 3T3-ShhFL cells either diluted to 6.25% or added without dilution to demonstrate that compounds tested were not metabolized by Caco-2 cells during the 6hr experiment. All medium tested were capable of inhibiting Hh pathway response even with dilution thus confirming the stability of each compound. Data show the mean and SD of three samples. Two independent experiments were performed.

Supplementary Figure S3

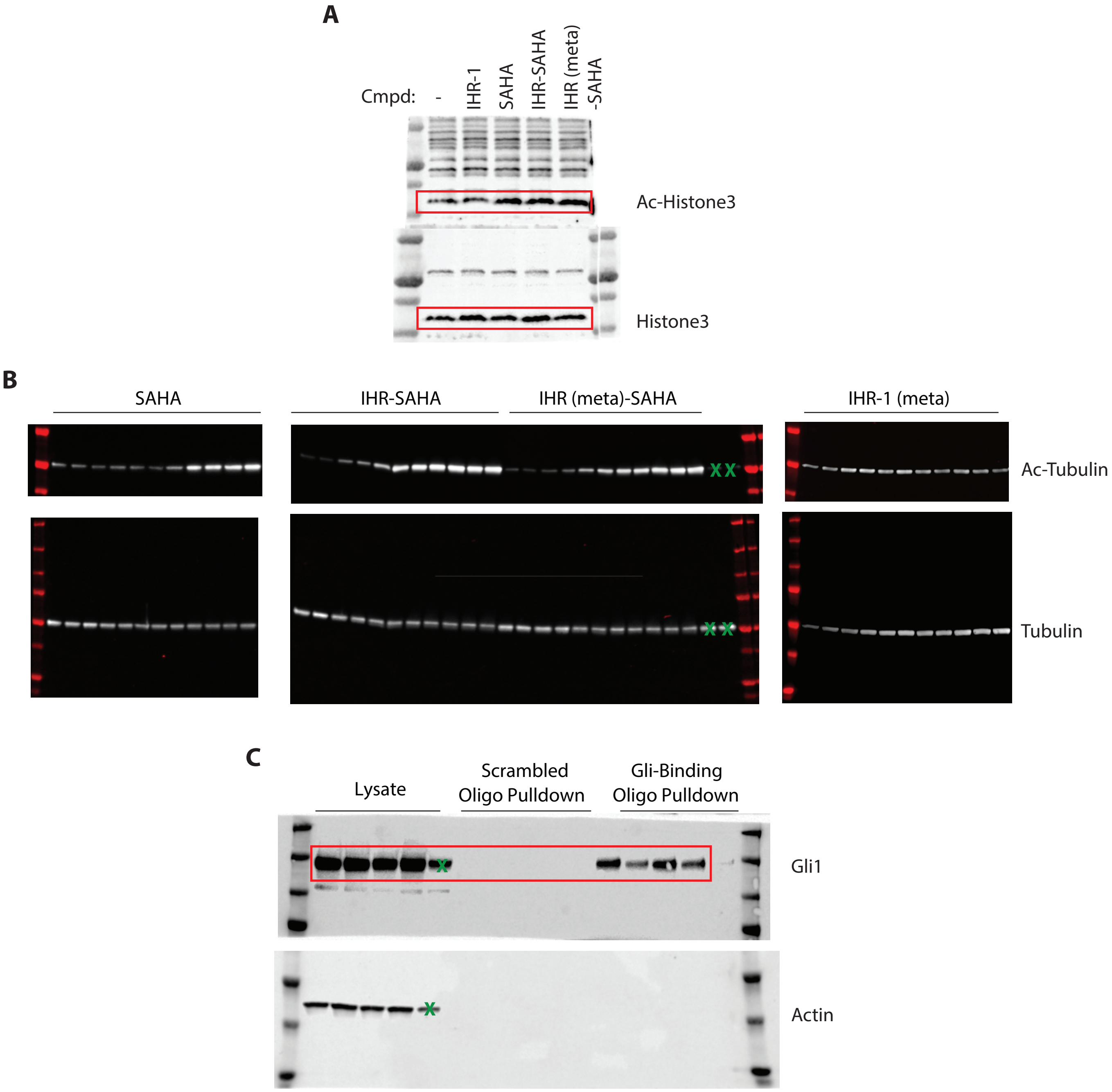

Supplementary Figure S3. Unprocessed Western blots. A. Acetylated histone3 for Fig. 3B. **B.** Acetylated tubulin for Fig. 5. **C.** Gli1 for Fig. 6B.
